# Optimizing prostate magnetic resonance imaging: toward smarter imaging pathways

**DOI:** 10.1590/0100-3984.2025.58.e3

**Published:** 2025-09-09

**Authors:** Fahd Alkhalifah, Jorge Abreu-Gomez, Adriano B. Dias

**Affiliations:** 1 University Medical Imaging Toronto, Joint Department of Medical Imaging, University Health Network–Sinai Health System–Women’s College Hospital, University of Toronto. Toronto, ON, Canada.

Magnetic resonance imaging (MRI) has become a cornerstone in the diagnosis and management of prostate cancer, with established roles in initial detection, active surveillance, and post-treatment assessment^(1^,^2)^. Multiparametric MRI (mpMRI)—which includes T2-weighted imaging (T2WI), diffusion-weighted imaging (DWI), and dynamic contrast-enhanced (DCE) imaging—has enabled more precise risk stratification, targeted biopsy, and treatment planning. However, as global demand for prostate MRI increases, concerns regarding scan duration, cost, and resource utilization are becoming more pressing. Biparametric MRI, which omits DCE-MRI, has shown comparable performance in certain populations, suggesting that it is feasible to take approaches that are more streamlined^(3^,^4)^. With rising case volumes, further optimization of MRI workflows is essential to balance diagnostic accuracy and clinical efficiency.

In this context, the pilot study conducted by Firoozeh et al.^(5)^, published in **Radiologia Brasileira**, offers timely and provocative insights. The authors assessed whether an early negative result on T2WI or DWI could reliably predict a completely negative mpMRI, potentially allowing early scan termination. In 492 scans compliant with Prostate Imaging Reporting and Data System, version 2.1, they found that the scan could have been completed after just one negative sequence in 33% of the patients with suspected cancer and in 10% of those with known cancer. They also found that DWI was superior to T2WI: 88.9% of negative DWI scans were followed by negative T2WI/DCE sequences, compared with 62.4% for negative T2WI scans predicting negative DWI/DCE sequences. These results held after adjustment for patient and reader variables.

This hypothesis-generating study mirrors commonly utilized decision-making algorithms in other imaging workflows, such as adrenal computed tomography^(6)^. The appeal is evident: if a substantial proportion of mpMRI scans are ultimately negative, early scan truncation based on real-time interpretation could improve access, reduce costs, and limit unnecessary imaging. Patients would benefit from shorter scan times and, when contrast is avoided, reduced exposure.

The concept of using DWI as a first-line or stand-alone sequence has already been explored in the context of prostate cancer diagnosis. Reijnen et al.^(7)^ recently showed that high-resolution monoparametric DWI achieved 100% sensitivity for clinically significant prostate cancer, suggesting that in a subset of patients, additional sequences may be unnecessary. Their findings complement and expand on those of Firoozeh et al.^(5)^, supporting the strong negative predictive value of DWI, particularly in low- to intermediate-risk populations. However, some limitations must be acknowledged. Although early-sequence prediction appears statistically sound, the clinical risk of prematurely terminating a scan remains non-trivial. In the Firoozeh et al. study^(5)^, 11% of negative DWI sequences were followed by positive findings in subsequent phases. In those cases, early termination could lead to underdiagnosis of clinically significant cancers—especially given the known pitfalls of each sequence when used in isolation^(8^,^9)^. In addition, the retrospective design and potential verification bias, despite mitigation attempts through re-review, limit the generalizability of these findings. Future validation should ideally involve blinded and sequential reading.

Operational barriers also exist. Requiring radiologists to interpret and act in real time would increase cognitive demands, introduce variability, and potentially slow workflow^(10)^. Artificial intelligence (AI) could eventually fulfill that role, assessing early sequences and determining whether further imaging is warranted. Although preliminary efforts in AI-based sequence optimization have shown promise^(11)^, broader validation is needed, especially given the variability of DWI acquisition across vendors and platforms.

Despite these challenges, the Firoozeh et al. study^(5)^ contributes to an emerging paradigm: personalized MRI protocols based on real-time imaging content and predictive modeling. Current guidelines emphasize standardization, whereas future directions may embrace adaptive protocols reflecting patient risk profiles and early imaging findings—similar to evolving approaches in computed tomography colonography and abbreviated breast MRI^(12)^. These abbreviated strategies are also aligned with the emerging role of MRI-based prostate cancer screening, in which the high negative value assurance of DWI may prove useful. Overall, protocols that are scalable and streamlined will be critical to manage the expected surge in demand^(13)^.

In conclusion, Firoozeh et al.^(5)^ present compelling early data suggesting a pathway to more efficient, intelligent prostate MRI. Although not yet ready for routine clinical practice, these findings set the stage for further investigation—particularly in conjunction with AI tools. As the role of prostate MRI continues to expand, such innovations will be essential to balancing diagnostic accuracy with operational feasibility in high-volume and resource-limited settings.

## References

[1] Chappidi MR, Lin DW, Westphalen AC (2025). Role of MRI in active surveillance of prostate
cancer. Semin Ultrasound CT MR.

[2] Dias AB, Chang SD, Fennessy FM (2025). New prostate MRI scoring systems (PI-QUAL, PRECISE, PI-RR, and
PI-FAB): AJR expert panel narrative review. AJR Am J Roentgenol..

[3] Twilt JJ, Saha A, Bosma JS (2025). Evaluating biparametric versus multiparametric MRI for diagnosing
clinically significant prostate cancer: an international, paired,
noninferiority observer study. Eur Urol..

[4] Dias AB, Moore CM, Renard-Penna R (2025). Biparametric versus multiparametric MRI in prostate cancer: a
choice or a fine balance?. Eur Urol..

[5] Firoozeh N, Behr SC, Westphalen AC (2025). Predicting uninformative prostate magnetic resonance imaging
sequences: a hypothesis-generating pilot study. Radiol Bras..

[6] Mayo-Smith WW, Song JH, Boland GW (2017). Management of incidental adrenal masses: a white paper of the ACR
incidental findings committee. J Am Coll Radiol..

[7] Reijnen JS, Ryg U, Marthinsen JB (2023). Monoparametric high-resolution diffusion-weighted MRI as a
possible first step in an MRI-directed diagnostic pathway. Front Oncol..

[8] Rosenkrantz AB, Taneja SS (2014). Radiologist, be aware: ten pitfalls that confound interpretation
of prostate mpMRI. AJR Am J Roentgenol..

[9] Moldovan PC, Van den Broeck T, Sylvester R (2017). What is the negative predictive value of multiparametric magnetic
resonance imaging in excluding prostate cancer at biopsy? A systematic
review and meta-analysis from the European Association of Urology Prostate
Cancer Guidelines Panel. Eur Urol..

[10] Patel AG, Pizzitola VJ, Johnson CD (2020). Radiologists make more errors interpreting overnight CT scans
versus daytime. Radiology..

[11] Shimron E, Perlman O (2023). AI in MRI: computational frameworks for a faster, optimized, and
automated imaging workflow. Bioengineering (Basel).

[12] Kuhl CK, Schrading S, Strobel K (2014). Abbreviated breast magnetic resonance imaging: first postcontrast
subtracted images and maximum-intensity projection—a novel approach to
breast cancer screening with MRI. J Clin Oncol..

[13] Schoots IG, Padhani AR (2025). A paradigm shift toward population-based MRI-targeted prostate
cancer screening. JAMA Oncol..

